# Battle of oral anticoagulants in the field of atrial fibrillation scrutinized from a clinical practice (the real world) perspective

**DOI:** 10.1186/1477-9560-9-12

**Published:** 2011-07-27

**Authors:** Raul Altman, Hector O Vidal

**Affiliations:** 1 Centro de Trombosis de Buenos Aires. Viamonte 2008, 1056 Buenos Aires, Argentina; 2 Hospital Italiano de La Plata, Area de Hematología, Sección Hemostasia y Trombosis, 1900 La Plata, Pcia. de Buenos Aires, Argentina

**Keywords:** Atrial fibrillation, Oral anticoagulants, Antiplatelet drugs, Aspirin, Dabigatran, Rivaroxaban, Apixaban

## Abstract

Warfarin has a long history of benefit and has become the gold standard medication for the prevention of ischemic stroke in patients with atrial fibrillation. Nevertheless, it is far from perfect and there is no doubt that new drugs must be found to replace warfarin. The new oral anticoagulants that are on the market or awaiting approval or under research offer some benefits but not enough to replace warfarin until results of additional studies can show an adequate balance between effectiveness/safety and cost/benefit. There are several issues concerning the new oral anticoagulants. It is essential that the effect of any anticoagulant can be measured in plasma. But to date, there is no test to assess the effect or therapeutic range for the new oral anticoagulants. There is no antidote to neutralize the action of the new drugs in cases of bleeding or when acute surgical intervention is necessary. Dabigatran requires dose adjustment in patients with moderate renal impairment and is contraindicated in patients with severe renal failure. Rivaroxaban should be used with caution in patients with severe renal impairment. Apixaban excretion is also partly dependent on renal function, although the impact of renal insufficiency has not yet been determined. How anticoagulant bridging can be done before surgery has not yet been established. In conclusion, although thousands of patients have been treated in phase III studies, additional data are necessary before conclusions can be drawn on the potential for these new anticoagulant drugs to replace warfarin in patients with atrial fibrillation.

## Introduction

Atrial fibrillation (AF) is the most common arrhythmia. AF can present with significant symptoms or with just a few cardiodynamic modifications that the patient is not aware of. Its most feared complication is embolization especially in the central nervous system. Each year, in the United States alone, AF causes more than 50,000 strokes [1]. US statistics show there are currently more than 2.3 million people with AF. This number is expected to reach 6 million by 2050 in the United States. Without adequate prophylactic and therapeutic measures, morbidity and mortality from thromboembolism will also increase in the future [2].

The pathophysiology of thrombosis indicates that under conditions of high blood flow, the participation of platelets in the initiation of a thrombus is the most important target for inhibitors of platelet function used as primary therapy. In the case of medium flow, anticoagulant drugs seem to be a more appropriate therapy. A combination of both strategies should not be ruled out to provide better prevention than each individual therapy. But during combined therapy, the potential benefits could be distorted by adverse effects caused by increased bleeding. Any antithrombotic drug or drug combination with a higher level of prevention is certainly potentially more hemorrhagic. This could be called the golden rule in antithrombotic therapy.

The standard therapy available for thromboembolic prevention in patients with AF is warfarin and in patients with low risk according to the CHADS2 scale, aspirin or no pharmacological therapy. Anticoagulant therapy cannot be indicated when it is not possible to control therapy due to difficulty in maintaining adequate international normalized ratio (INR) values (2.0-3.0), reluctance of patients to undergo frequent blood tests, or due to the risk factors that predispose to bleeding, etc. The reality is that only 50-60% of patients with AF who are suitable for anticoagulant therapy receive it preventively [3].

Recently, new antithrombotic drugs have become available or are in phase III of clinical research, and after more than 50 years will compete with warfarin in the field of AF prevention. Warfarin has been, without doubt, the gold standard medication for the prevention of ischemic stroke but there are several reasons why this medication is far from perfect. On the one hand, it has the benefit of a well-established efficacy, there is a specific antidote in case of bleeding and the need to discontinue the medication in an urgent situation, it has no side effects and is not expensive. On the other hand, warfarin has several disadvantages. Frequent monitoring is required to maintain the INR between 2.0 and 3.0 which, even in the best hands, is achieved in 55-60% of patients. It is necessary to have specialized clinics and the therapeutic window is narrow. Warfarin has many interactions with food and medicine, has a long half-life and a very slow onset of action, and its pharmacokinetic profile is affected by genetic polymorphisms that make patients respond inadequately to medication. Moreover, although the prothrombin time assay is a simple test, INR (the test of choice for controlling warfarin) standardization outside specialized laboratories is difficult. During surgery or other procedures, interruption of treatment requires therapeutic tactics in skilled hands.

There are patients who need not be subjected to anticoagulation. How can they be identified? If anticoagulation is unavoidable, which patients will benefit? The consensus of the European Society of Cardiology recommended using the CHADS2 risk score; when it is 0, no medication or aspirin is indicated. When the risk score is 1, the use of aspirin or warfarin is appropriate and medical criteria will define which one. When the risk score is ≥2, oral anticoagulation is indicated. The CHA2D2-VASc scale [4] adds additional risk factors that may be useful especially in patients with a risk score of one to decide between the use of anticoagulants or aspirin.

The most severe complication that can arise from the use of anticoagulants is bleeding. It should be emphasized that not only severe hemorrhage that affects the brain, kidneys, or gastrointestinal tract is important. When minor bleeding occurs, medication is stopped, placing the patient in a potentially prothrombotic state.

It must accepted that any drug that has a higher level of prevention will have potentially higher hemorrhagic risk; this clinical problem is still unresolved. Therefore it is essential that the effect of any anticoagulant medication can be measured in plasma, as done by prothrombin time for warfarin control, activated partial prothrombin time for administration of regular unfractionated heparin or the assay for plasma levels of anti-activated factor X in the case of low molecular weight heparins to avoid excessive or deficient anticoagulation predisposing to hemorrhagic or thrombotic accidents.

Among the potential new therapies a warfarin-related drug, tecarfarin [5], a vitamin K antagonist similar to warfarin, has been studied. It is a vitamin K epoxide reductase inhibitor which decreases the activity of vitamin K-dependent coagulation factors (factors II, VII, IX, and X) and prolongs the prothrombin time [6]. The advantage is that it is metabolized through an esterase, is not metabolized by cytochrome P450 [5] and avoids the many interactions with food and medicine of warfarin. Its control can also be monitored by the prothrombin time and expressed as the INR, which seems to remain more stable than with warfarin. The first study was published in *Circulation *2 years ago [7] on a group of 66 patients, and more trials can be expected to identify further benefits compared with warfarin and other oral anticoagulants. Having a specific antidote and a more stable action compared with warfarin gives tecarfarin an interesting profile for the prevention of thromboembolic diseases.

## Analysis of antithrombotic strategies in AF

### Oral anticoagulants and antiplatelet drugs

Before discussing studies on thromboembolic prevention in AF, it must be borne in mind that patients seen in daily clinical practice (the real world) often do not fit the profile of those included in clinical trials.

Patients with AF have a 5-fold higher incidence of ischemic brain injury and increased mortality. For several decades, warfarin has been shown to be the medication of choice for the prevention of thromboembolism in these patients. In 1994 a group of 3691 patients included in 5 studies with and without treatment with warfarin showed 68% risk reduction obtained by anticoagulant therapy, with virtually no increased risk of bleeding [8]. Pooled analysis of patient-level data from six published randomized clinical trials comparing aspirin with warfarin showed that warfarin significantly reduced the rate of ischemic stroke compared with aspirin [9]. Also in 2007, a meta-analysis from 29 trials that included 28,044 participants showed that warfarin improved outcomes by 40% compared with antiplatelet therapy in patients with AF [10].

Warfarin was found to be more protective than aspirin even though those studies did not take into account risk levels [11], but benefit was obtained even in patients older than 75 years (mean 81.5 years) [12].

More recently, after the widespread use of clopidogrel in cardiology, it has been suggested that warfarin can be replaced with the combined use of aspirin + clopidogrel. We consider this possibility rational as we reported that this antiplatelet drug combination lowered the amount of thrombin formed in a system in vitro [13].

The ACTIVE study compares aspirin + clopidogrel with warfarin and clopidogrel + aspirin with aspirin alone [14]. The results (Table 1) indicate that warfarin is superior to the combination of clopidogrel + aspirin in the prevention of vascular events (*P *= 0.0002; number need to treat [NNT] = 50) with no increased incidence of major bleeding (*P *= 0.67; number need to harm [NNH] = 476).

**Table 1:** Active W Study: outcomes

Outcomes	Clopidogrel + aspirin	Warfarin	NNT or NNH	*P*
Vascular events (%/year)	5.64	3.63	50	0.0002
Major bleeding (%/year)	2.42	2.21	476	0.67

NNH, number needed to harm; NNT, number needed to treat.

Moreover, the use of clopidogrel associated with aspirin (study Active A) prevented more thromboembolic events than aspirin alone but at the expense of a significant increase in major bleeding, and with a tendency to increased mortality. As clopidogrel plus aspirin reduces the risk of major vascular events, this combination is indicated when treatment with warfarin is difficult because patients refuse to be monitored or where controls cannot be done or are not reliable. In this regard, the possibility of resistance to clopidogrel and/or aspirin [15,16] should be investigated.

In the AVERROES study [17], apixaban, an oral direct inhibitor of activated factor X in doses of 5 mg twice per day, was compared with aspirin. In this study, apixaban was administered to 5600 patients with AF who had relatively low risk (level 1-2 as measured by the CHADS2 scale) and could not be medicated with warfarin. Apixaban was compared with aspirin 81-324 mg/day. The study was stopped ahead of schedule due to the benefit seen in patients with apixaban. The reduction of ischemic stroke was statistically significant (*P *< 0.001) without increasing major bleeding complications and a slight increase in minor bleeding (NNT = 91),

### Warfarin and the new oral anticoagulants

Table 2 shows some pharmacodynamic characteristics of the newer antithrombotic compounds compared with warfarin. Of the new medication, only dabigatran has been approved for use in AF. The other drugs are in phase III studies. Trials designed to compare the new agents with warfarin (Table 3) and based on the criterion of noninferiority, have shown a significant effect in the prevention of thromboembolic complications in patients undergoing orthopedic surgery. Will these new anticoagulants have a real impact on thromboembolic prevention, especially stroke, in patients with AF? After presenting comparative studies in the following paragraphs, the advantages and disadvantages in relation to warfarin are discussed.

**Table 2:** Characteristics of new oral anticoagulants compared with warfarin

Agent	Main action	Half-life (h)	Renal clearance (%)	Cross placenta	Dose	Interactions
Rivaroxaban	Anti-factor Xa	6-10	66	+	Once daily	CYP3A4 inhibitors
Apixaban	Anti-factor Xa	10-15	30	+	Twice daily	CYP3A4 inhibitors
Dabigatran	Anti-factor IIa	12-14	80	+	Twice daily	PPIs
Warfarin	Synthesis of vitamin K-dependent factors	36-50		+	Adjusted by INR, once daily	Multiple with food and drugs

PPIs, proton pump inhibitors. Quinidine is contraindicated for patients on dabigatran. Amiodarone or rifampicin require caution.

**Table 3:** Trials designed to compare the new oral anticoagulants to prevent thromboembolism with warfarin in AF

Trial	Study drug	Dosing	Number of patients	Design
RE-LY	Dabigatran	110 mg twice daily, 150 mg twice daily	18,113	Randomized, open-label, noninferiority
Rocket-AF	Rivaroxaban	15 mg daily, 20 mg daily	14,000	Randomized, double blind, noninferiority
ARISTOTLE	Apixaban	5 mg twice daily	15,000	Randomized, double blind, noninferiority
Engage AF	Edoxaban	30 mg daily, 60 mg daily	16,500	Randomized, double blind, noninferiority

Dabigatran etexilate is a prodrug that becomes the active principle dabigatran with specific inhibiting effects of thrombin both free and bound to fibrin. In the RE-LY study (Randomized Evaluation of Long-term anticoagulant therapY) [18,19] dabigatran was administered in two dosages: 150 mg or 110 mg twice daily. The results based on the criterion of noninferiority indicate that the dosage of 150 mg twice a day was significantly more effective than warfarin in the prevention of ischemic stroke with similar frequency of hemorrhagic stroke. The dosage of 110 mg twice a day was similar to warfarin in the prevention of thromboembolism and presented with lower hemorrhagic events. Patients treated with a dosage of 150 mg twice daily had a 35% reduction in systemic embolism and 74% of the risk of hemorrhagic stroke. These numbers are impressive.

The NNT can describe results from the perspective of daily medical practice (Table 4). Although the differences between dabigatran and warfarin in some of the outcomes are significant and related to the number of patients included (6000 per group), the NNT of the end points are unconvincing and the 35% reduction in stroke does not seem as impressive. Results from phase IV studies would provide more data on efficacy and safety ratios.

**Table 4:** Outcomes with dabigatran compared with warfarin

End points	Dabigatran 110 mg ×2	Dabigatran 150 mg ×2	Warfarin	NNT/NNH: dabigatran 110 mg	NNT/NNH: dabigatran 150 mg
Primary outcomes (%)	1.53	1.11	1.69	625	172
Myocardial infarction (%)	0.7	0.7	0.5	500	500
Mortality (%)	3.8	3.6	4.1	330	200
Major bleeding (%)	2.7	3.1	3.4	143	333
Intracranial bleeding (%)	0.2	0.3	0.7	200	250
Net clinical benefits (%)	7.1	6.9	7.6	200	143

NNH, number needed to harm; NNT, number needed to treat. Net clinical benefits: vascular events, death and major bleeding. Data from the RE-LY trial [16].

When the side effects are considered (see below), it is perhaps still premature to advocate this medication. For example, the end points do not take into account minor bleeding, which, although it does not complicate the clinical evolution of patients, can result in the suspension of medication and a transient prothrombotic state. Moreover, patients in the dabigatran group discontinued the medication in larger numbers than those with warfarin, because of gastrointestinal symptoms. Myocardial infarction was also was more common in patients treated with dabigatran [19].

In certain circumstances (e.g., in patients with AF and with coronary stent), the triple combination of aspirin, clopidogrel and oral anticoagulants is required. Oldgren et al. [20] compared triple therapy with dabigatran in patients with recent myocardial infarction. Their study showed that 3.8% of patients taking placebo died or had a heart attack or stroke compared with dabigatran at different doses, twice daily; 4.6% in those treated with 50 mg, 4.9% for 75 mg; 3.0% for 110 mg and 3.5% for 150 mg. Hemorrhages (major or clinically relevant minor bleeding) during the 6-month treatment period increased dose-dependently with dabigatran: the hazard ratio was 1.77 for 50 mg, 2.17 for 75 mg, 3.92 for 110 mg, and 4.27 for 150 mg compared with placebo.

It is interesting that the US Food and Drug Administration (FDA) approved the 150 mg twice daily dosage but not the lower dose and instead approved a 75 mg twice daily dosage for patients with renal insufficiency with creatinine clearance less than 30 mL/min. This is supported by the Oasis 6 study [21], in which a statistically significant increase in bleeding was observed in patients with creatinine clearance ≤30 mL/min when using enoxaparin.

To investigate 110 mg dose, Eikelboom et al. [22] compared hemorrhagic stroke in patients from the RE-LY study who were older and younger than 75 years and found that both doses of dabigatran have lower risks of both intracranial and extracranial bleeding in patients aged <75 years compared with warfarin. In those aged ≥75 years, intracranial bleeding risk was lower but extracranial bleeding risk was similar or higher with both doses of dabigatran compared with warfarin. This means that the positive balance of dabigatran is less evident in older patients. The safety advantages of dabigatran compared with warfarin are less evident with increasing age.

Rivaroxaban is a new oral anticoagulant drug that acts by inhibiting activated factor X. The Rocket-AF study [23] compared rivaroxaban with warfarin in patients with AF. It included more than 14,000 patients in a noninferiority designed trial. Rivaroxaban dosage was 15-20 mg/day and warfarin planned to maintain an INR of 2.0-3.0. The primary end point was a reduction in embolic events and evaluation of bleeding complications.

The same criteria as for dabigatran can be applied with regard to the NNT (Tables 5 and 6). For some primary outcomes where the difference with warfarin is significant *P *< 0.001), at least 192 patients must be treated in daily practice to prevent 1 case of vascular death, stroke, or embolism.

**Table 5:** Rocket-AF study: primary ischemic outcomes

Outcomes	Rivaroxaban	Warfarin	*P*	NNT
Primary outcomes (noninferiority)^a^	1.71	2.16	<0.001	222
Primary outcomes (on treatment)	1.70	2.15	0.015	222
Non-central nervous system embolism	0.04	0.19	0.003	667
Vascular death, stroke, embolism	3.11	3.63	0.034	192
Ischemic stroke	1.34	1.42	0.581	1250
Unknown cause	0.06	0.10	0.366	2500

NNT, number needed to treat. Data from Mahaffey KW. AHA Scientific Sessions 2010.

^a^Stroke and extracranial embolism, event rate per 100 patients/year.

**Table 6:** Rocket-AF study: primary safety outcomes

Outcomes	Rivaroxaban	Warfarin	*P*	NNT/NNH
Major and non-major clinically relevant	14.91	14.52	0.442	333
≥2 g/dL Hb drop	3.60	3.45	0.576	667
Transfusion (>2 U)	2.77	2.26	0.019	196
Critical organ bleeding	0.82	1.18	0.007	278
Bleeding causing death	0.24	0.48	0.003	417
Hemorrhagic stroke	0.26	0.44	0.024	556
Intracranial hemorrhage	0.49	0.74	0.019	400

NNH, number needed to harm; NNT, number needed to treat. Data from Mahaffey KW. AHA Scientific Sessions 2010.

The study results showed that rivaroxaban significantly reduced intracranial bleeding compared with warfarin. With regard this safety point, between 278 and 417 patients must be treated to obtain 1 case of reduction in critical organ bleeding or bleeding causing death or intracranial hemorrhage in favor of rivaroxaban.

The MAGELLAN study [24] (data from A. Cohen presented at the American College of Cardiology Scientific Sessions 2011) is an approach on security in nonsurgical patients and serves to maintain alert about the hemorrhagic possibilities. Eight thousand one hundred and one patients were randomized to 10 mg rivaroxaban once daily for 35 days or standard treatment with subcutaneous enoxaparin 40 mg once a day for 10 days. The results of the MAGELLAN study show that when rivaroxaban was administered for 35 days to prevent deep venous thrombosis (composite efficacy outcomes at day 10; 2.7%), there were no differences between rivaroxaban and enoxaparin; at day 35, NNT = 76.9 (rivaroxaban 4.4% enoxaparin 5.7%) with the following increased bleeding complications: clinical relevant bleeding at day 1-10 NNH = 62.5 (rivaroxaban 2.8%, enoxaparin 1.2%); at day 11-35 NNH = 111 (rivaroxaban 1.4%, enoxaparin 0.5%). The rational question is whether these results can be assimilated to what might happen in patients with AF who are under treatment for much longer periods. This requires taking into account certain characteristics of the MAGELLAN study (the comparison was made with enoxaparin; the clinical patients apparently had more severe disease and high mortality rate), but nevertheless this indicates again that a fixed dose without laboratory control leads to a negative balance in efficacy/safety for new antithrombotics.

Apixaban, another direct inhibitor of activated factor X, was also used to assess benefit in patients with AF [25]. The ARISTOTLE study is similar to the AVERROES study already mentioned above. Apixaban was used at a dose of 5 mg twice daily. As with other oral antithrombotics, the comparator was warfarin and more than 18,000 patients were included. Definitive data have not yet been published.

The efficacy/safety ratio of apixaban was recently published in the APPRAISE-2 study, in a different population and added to antiplatelet therapy [26]. APPRAISE-2 trial included patients who were at high risk following acute coronary syndrome. Patients were on antiplatelet therapy and were randomized to either placebo or two 5-mg daily doses of apixaban. After enrolling 7392 patients trial was stopped because data showed an increase of intracranial and fatal bleeding events in the apixaban group than the placebo group and the primary end point of cardiovascular death, MI, or ischemic stroke were similar in both groups. Could control of anticoagulant effect of apixaban leads to a positive balance in efficacy/safety?

Are there differences between the new drugs and their efficacy/safety ratios that gives one an advantage over the others? Taking into account data from the studies mentioned so far, there were differences in patients enrolled in the RE-LY [18], Rocket-AF [23] and ARISTOTLE [25] studies. Patients in the ARISTOTLE study accounted for a large population at risk, from CHADS2 risk score 1 to the highest risk scores. In the RE-LY study the risk score according to CHADS2 was moderate to mild (32% of patients at risk CHADS2 = 3-6) and the Rocket-AF study included patients with moderate to severe risk (87% of patients at risk CHADS = 3-6) which will make comparisons difficult, even when definitive data are available.

Other oral antithrombotic drugs on which no data are available yet are Edox (Daiichi-Sankyo), TAK-442 (Takeda), Betrix (Portola/Merck), and Darex (Astellas), all of which have been developed for the prevention and treatment of deep vein thrombosis.

## Adverse effects

As mentioned earlier in this article, we consider as axiomatic that a drug that improves efficiency will potentially be accompanied by an increase in bleeding (the golden rule). The studies generally show that increased prevention is accompanied by an increase in major or minor bleeding complications. The careful choice of patients and assessment of bleeding risk using the HAS-BLED score [27] can help in the selection. When a laboratory assay is established to determine the degree of anticoagulation as well as the therapeutic range of any new drug, it is likely that direction can be adjusted to raise its profile and then advise warfarin replacement.

In the RE-LY study, patients had more dyspepsia probably caused by the low pH of the medication. This resulted in increased drug discontinuation compared with warfarin.

Another side effect is the increased risk of myocardial infarction. This paradoxical effect, seen very marginally in the RE-LY study, has already been reported in REEDEM [20], a phase II study on patients with acute coronary syndrome and also noted with the use of a related drug, ximelagatran. This may be due to the pharmacology of dabigatran (level of inhibition of thrombin) or just because there are studies showing that warfarin protects patients from myocardial infarction.

The possibility of myocardial infarction does not seem to occur with the use of rivaroxaban but ongoing studies are required to demonstrate its efficacy in the prevention of acute coronary syndromes [28].

Before use of these drugs, renal function should be established and monitored because in the presence of renal function impairment, the dosage of dabigatran must be adjusted or stopped.

## Disadvantages of dabigatran (some are also applicable to rivaroxaban and apixaban)

1. The administration of two doses daily, which favors forgetfulness by the patient.

2. Forgetting more than one dose can put the patient at a prothrombotic risk.

3. Gastric intolerance leading to stopping the medication.

4. The possibility, although low, of myocardial infarction.

5. There is no antidote to neutralize the action of the dabigatran in cases of bleeding or when acute surgical intervention is necessary.

6. The anticoagulant effect must be controlled and there is no test to assess the effect or levels of therapeutic range.

7. Caution is advised in the case of impaired renal function. Dosing should be reduced in renal failure or the medication discontinued. Nevertheless there are no studies that clearly indicate the dose to use in these circumstances.

8. In phase III studies, patients have shown a small increase in hemorrhages; data related to this risk must be corroborated in phase IV.

9. The instability of the drug once the packaging is opened.

10. There are some drug interactions that must be known (see Table 6).

11. As for other anticoagulants, age is an additional risk factor for hemorrhages.

12. It is difficult to validate patient compliance.

13. How anticoagulant bridging can be done before surgery has not yet been established.

14. Cost.

Points 5-8 and 10-14 also apply to rivaroxaban and apixaban. Rivaroxaban should be used with caution in patients with severe impaired renal function. Apixaban excretion is partly dependent on renal function, although the impact of renal insufficiency has not been determined.

## Advantages of dabigatran, rivaroxaban and apixaban

1. Fixed dose.

2. No laboratory control (strong arguable).

3. Few drug interactions.

4. No food interactions.

## Drawbacks of warfarin

1. Warfarin requires frequent monitoring to maintain the INR between 2.0 and 3.0 and this is achieved, at best, in only 55-60% of patients.

2. The therapeutic window is narrow.

3. Its onset of action is slow and, according to the basal values of vitamin K factors, between 3 and 6 days are needed to reach therapeutic levels.

4. There are many interactions with medications and meals.

5. Polymorphisms exist that could determine increased sensitivity or resistance to warfarin.

6. The suspension of the medication before a surgical procedure is difficult.

7. Warfarin has a very long half-life.

8. Prothrombin time with INR determination is the best method available to control therapy but is not good enough.

9. Specialized centers are required for its control.

10. Age is an additional factor in the risk of bleeding.

## Advantages of warfarin

1. Well-established efficacy.

2. Adequate efficacy/safety ratio.

3. Its effect can be reversed by vitamin K.

4. Very low cost.

5. No side effects.

## Conclusions

Warfarin has many disadvantages as an anticoagulant with a long history of benefits. There is no doubt that new drugs must be found to replace warfarin. The new oral anticoagulants that are on the market or awaiting approval or further research offer some benefits but still cannot replace warfarin until phase IV results show a proper balance between effectiveness and safety, and cost/benefit.

Should patients who are on oral anticoagulation with warfarin be switched to the new oral antithrombotics?. The answer depends, as indicated in the text, on the conditions under which the anticoagulant effect of the new drugs can be controlled and after determining their therapeutic levels. Nevertheless, if patients are stable within a therapeutic INR, they should remain on warfarin.

The combined use of dual antiplatelet therapy with warfarin is mandatory in certain thrombotic risk situations. In patients with AF, warfarin, aspirin, and clopidogrel are associated with more than 3-fold increased risk of nonfatal and fatal bleeding [29] and this will probably also apply with the new drugs, both new anticoagulants and new antiplatelet drugs. These strategies determine the potential increase in severe or moderate hemorrhagic events and even life compromise. There are insufficient studies to recommend strategies on this point [29-31]. Proton pump inhibitors could probably help in preventing gastric hemorrhage [32].

## Competing interests

The authors declare that they have no competing interests.

## Authors' contributions

RA Acquired the data, analyzed and interpreted the data, drafted the manuscript, made critical revision of the manuscript.

HOV Analyzed and interpreted the data, drafted the manuscript, made criticalrevision of the manuscript

All authors read and approved the final manuscript
